# Preantral follicle population and distribution in the horse ovary

**DOI:** 10.1530/RAF-21-0100

**Published:** 2022-04-04

**Authors:** Kendall A Hyde, Francisco L N Aguiar, Benner G Alves, Kele A Alves, Gustavo D A Gastal, Melba O Gastal, Eduardo L Gastal

**Affiliations:** 1 Animal Science, School of Agricultural Sciences, Southern Illinois University, Carbondale, Illinois, USA; 2 Department of Veterinary Medicine, Sousa Campus, Federal Institute of Education, Science and Technology of Paraíba, Sousa, Paraíba, Brazil; 3 Instituto Nacional de Investigación Agropecuaria, Estación Experimental INIA La Estanzuela, Colonia, Uruguay

**Keywords:** equine ovarian plasticity, preantral follicles, spatial distribution and population, folliculogenesis

## Abstract

Characterization of the ovarian preantral follicle population is a necessary step to improve understanding of folliculogenesis and ovarian physiology. Therefore, in the present study, the preantral follicle population in the equine ovary in young and old mares was investigated according to follicular morphology, follicular class, distance from the geometric center using ovarian maps, and follicular density within ovarian portions (lateral vs intermediary) and regions (dorsal vs ventral). Ovaries were collected from an abattoir and histologically processed for evaluation, and the follicle population was calculated. Overall, in the current detailed study, a higher preantral follicle population per mare ovary (mean: 82,206 ± 50,022; range: 1477 to 773,091) than originally reported was identified. Additionally, a mare age effect was observed in the follicle population (young: 152,664 vs old: 11,750) and the spatial distribution of morphologically normal and abnormal follicles and the density and population of follicular classes. These results demonstrate that, in addition to the preantral follicle population in the mare ovary being comparable to that of other species, the location and spatial distribution of these follicles is dynamic and varies depending on mare age and follicle status (i.e. morphology and developmental stage). The characterization of the distribution and population of preantral follicles in the mare ovary provided by this study can potentially aid in improving reproductive studies and assisted reproductive techniques and may expand the understanding of mechanisms involving ovarian plasticity and follicular migration.

**Lay summary:**

Knowledge of the distribution and population of immature eggs within follicles (preantral follicles) in the ovaries of mares can improve approaches to assisted reproductive techniques and fertility preservation. As the existing research on horse preantral follicle population was focused solely on large follicles, the present study provides an updated investigation of small and large preantral follicles in the mare, showing that the population is similar to those in other species. This study also shows that the way these follicles are distributed in the ovary varies depending on age and follicle characteristics. Results from this study may help to highlight which areas of the mare ovary should be looked at to find samples of good-quality follicles.

## Introduction

At birth in most species, a finite pool of preantral follicles exists in the ovaries of females (Kezele *et al.* 2002), which characterizes the main oocyte reserve of a given individual. Studies aiming to characterize the population of preantral follicles in the ovary are of great scientific value, as these can aid in increasing physiological knowledge about folliculogenesis, a critical concept for optimizing female fertility treatments and assisted reproductive techniques (ARTs).

In this context, the mare is a particularly appealing model to study folliculogenesis and follicle population, due to shared similarities with both women and other livestock species, making the mare a valuable dual-purpose, dual-benefit animal model (for review, see Carnevale 2008, Mihm & Evans 2008, Ginther 2012, Carnevale *et al.* 2020, Gastal *et al*. 2020, Benammar *et al.* 2021). Studies assessing preantral follicle population have been conducted in jennies (Lopes *et al.*2017), ewes (Amorim *et al.* 2000), does (Lucci *et al.*1999), cows (Lucci *et al.* 2002, Silva-Santos *et al*. 2011), gilts (Alves *et al.* 2012), and women (Gougeon & Chainy 1987). In mares, however, the only study in which follicular population was assessed counted preantral follicles (mean per ovary: 35,000; range per ovary: 6400–75,200) greater than 50 µm in diameter in young (2–4 years) mares (Driancourt *et al.* 1982). Nevertheless, it has been shown in recent studies that equine primordial, transitional, and primary preantral follicles have diameters smaller than 50 µm (Haag *et al*. 2013, Alves *et al.*2015). Thus, the original work assessing equine preantral follicle population (Driancourt *et al.* 1982) may have underestimated the number of follicles per ovary and, therefore, warrants an in-depth, updated study.

In addition to quantification of the follicular population in the mare ovary, the effects of age and supportive techniques used to evaluate preantral follicles (i.e. follicular morphology and classification, spatial distribution, and density) should simultaneously be characterized. The complex events of follicular development and migration are not uniform within the ovary (Riley *et al.* 2001, Faire *et al.* 2015). This leads to heterogeneity of the follicular population and large variation in the numbers and classes of follicles harvested in different samples of ovarian tissue in the mare (Alves *et al.*2016, Gastal *et al.*2017*a*) and several other species (woman: Schmidt *et al*. 2003, Kristensen *et al*. 2011; cow: Silva-Santos *et al.* 2011; ewe: Fransolet *et al.* 2014; doe: Brandão *et al.*2018; deer: Gastal *et al.* 2017*b*; mouse: Dath *et al.*2010, Malki *et al.*2015). This follicular heterogeneity and variation can be explained by the dynamic ovarian plasticity that has been suggested to occur in women and mares (Woodruff & Shea 2011, Alves *et al*. 2018). In fact, Alves *et al.* (2018) evaluated ovarian portions (lateral and intermediary) and regions (dorsal and ventral) in detail, considering the spatial distribution of preantral follicles according to mare age and follicle class. However, the morphology of preantral follicles across the whole ovary in different regions and portions according to mare age has not been evaluated. Thus, a novel study that combines an in-depth characterization of the follicle population with the distribution of morphologically normal follicles according to mare age is crucial.

The aims of this study were to assess the population of equine preantral follicles in young and old mares according to (i) follicular morphology, (ii) follicular class, (iii) distance from the ovarian geometric center, and (iv) follicular density within ovarian portions (lateral vs intermediary) and regions (dorsal vs ventral).

## Materials and methods

### Ovaries

**Figure 1:**
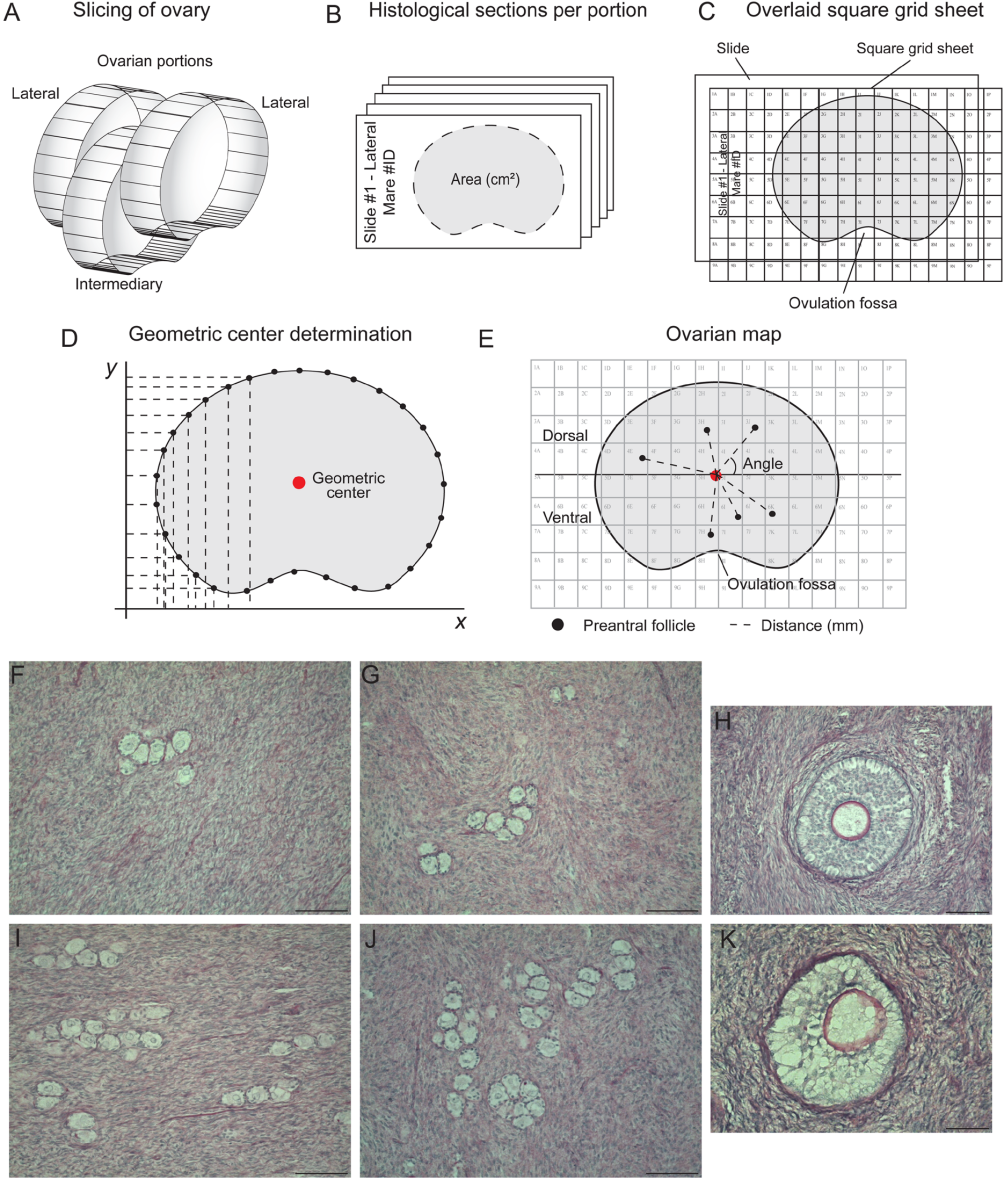
Illustration of experimental procedures performed to assess preantral follicle morphology, classification, spatial distribution, density, and population in the equine ovary. Ovaries were divided into (A) three portions (lateral, *n*  = 2; intermediary, *n*  = 1), followed by (B) histological processing and sectioning to allow for evaluation of follicle density and population. The whole follicle population was estimated per ovary using a formula, as previously described (Gougeon & Chainy 1987, Gastal *et al.* 2017*b*). (C, D, and E) A square grid sheet with columns and rows represented by letters and numbers, respectively, was placed upon the histological slides for microscopic evaluation. (D) Geometric center was determined for each histological section. (E) Analysis of preantral follicle distribution (distance in mm and angle in relation to the geometric center) and determination of dorsal and ventral regions of the ovary; five representative histological sections per ovarian portion per mare, now termed ‘ovarian maps’ (*n*  = 5 maps per portion; 15 maps per ovary; 240 maps total for all 16 ovaries from 8 mares), were made. Representative histological sections depicting (F and G) lower and (I and J) higher follicle (primordial, transitional, and primary) density per microscopic field are shown in low magnification. (H) Normal and (K) abnormal late secondary follicles are shown in high magnification. (F, G, I, and J) Scale bars = 100 µm, magnification = ×200; and (H and K) scale bars = 50 µm, magnification = ×400. (A–E) Diagrams adapted from Alves *et al.* (2018).

Ovaries were harvested during the physiological breeding season from mixed-breed, light-horse mares (*n*  = 8) at an equine abattoir located in Brazil (30°20’38”S, 54°20’31”W) and separated into two age groups (young: 4–9 years and old: ≥20 years; *n*  = 4 pairs of ovaries for each group) based upon dental characteristics. Immediately after slaughter, each ovary was divided into three longitudinal portions: *n*  = 2 lateral portions and *n*  = 1 intermediary portion (Fig. 1A). Afterward, each ovarian portion was immediately fixed in 4% paraformaldehyde for 24 h and placed in 70% alcohol until histological processing. None of the ovaries from the eight mares contained visible preovulatory follicles and/or corpora lutea. Reproductive status (anestrus or cycling) of the mares was unknown.

### Histological processing

All ovarian portions were dehydrated, embedded in paraffin wax, and completely cut into 7 μm serial sections (Alves *et al*. 2015). To avoid double counting of follicles, and considering the frequency and diameter of equine primordial and transitional follicles as reported by Alves *et al.* (2015), every fifth section of the ovary was mounted onto large (127 × 102 mm) microscope slides (Fig. 1B). To ensure good tissue quality for the analyses, only histological sections with clearly visible borders and intact ovulation fossa without lacerations were chosen. Slides were stained using periodic acid-schiff and counterstained with hematoxylin, then prepared for spatial distribution evaluation.

### Preparation for preantral follicle spatial distribution evaluation

The preparation of slides for determination of the spatial distribution for preantral follicles in the ovarian portions and regions was performed as previously described (Alves *et al.* 2018):

1. A square grid sheet (area of each square = 0.0625 cm^2^) with rows (indicated by numbers) and columns (indicated by letters) was designed and printed on an overhead transparency sheet at the same dimensions of the microscope slides.
2. Histological sections were overlaid with the square grid sheet (Fig. 1C) and scanned using a photo editing program (Adobe Photoshop CS4; San Jose, USA). All histological sections were scanned with the ovulation fossa positioned at the bottom and the square grid sheet aligned to the upper-left corner of the slide. These scanned, digital images were used as locating guides for the microscopic evaluation of the spatial distribution of preantral follicles.
3. Subsequently, the geometric center of each digitally scanned histological section was defined. Thirty equidistant points throughout the perimeter of each histological section were determined (Adobe Photoshop CS4; Fig. 1D). The distance of each point relative to the X- and Y-axes was recorded, and the geometric center was calculated using the following formulas:

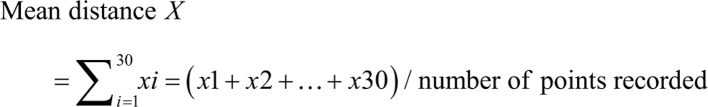

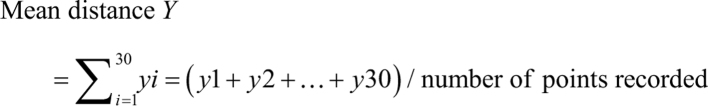

  Geometric center = mean distance *(X;Y)*
4. After determining the geometric center of each histological section, a longitudinal line was made using a marker tool (Adobe Photoshop CS4), and the ovarian regions above and below the longitudinal line were termed the dorsal and ventral regions, respectively.
5. Finally, five representative histological sections, from now on referred to as ‘ovarian maps’, per portion per ovary for each mare were used to assess preantral follicle distance from the geometric center (Fig. 1E).

### Microscopic evaluation

The histological sections were analyzed using light microscopy (Nikon E200; Tokyo, Japan) at ×400 magnification using an image capture system (Leica Imaging Software). For each ovarian portion, the following end points were evaluated considering preantral follicles: morphology, class, spatial distribution (concerning the ovarian region and distance from the geometric center), density, and population.

### Preantral follicle morphology and classification

Regarding morphology, preantral follicles were classified as normal (oocyte nucleus showing no signs of pyknosis, and the ooplasm surrounded by well-organized granulosa cells) or abnormal (oocyte showing a pyknotic nucleus or a retracted ooplasm with detachment or disorganization of the granulosa cells), as previously described (Alves *et al.* 2015; Fig. 1H and K). Only preantral follicles with a visible oocyte nucleus were counted and classified according to developmental class as primordial (oocyte surrounded by a single layer of flattened granulosa cells), transitional (oocyte surrounded by a single layer of both flattened and cuboidal granulosa cells), primary (oocyte surrounded by a single layer of cuboidal granulosa cells), or secondary (oocyte surrounded by two or more layers of cuboidal granulosa cells), as previously described (Alves *et al.* 2015).

### Measurement of distance from geometric center

Distance (mm) and angle (0º–360º) of the preantral follicles within each ovarian map were measured in relation to the geometric center using a ruler tool in the imaging software (Adobe Photoshop CS4). Polar plots using distance from the geometric center and angle (r, θ) coordinates were generated, as performed by Alves *et al.* (2018).

### Density determination

Regarding preantral follicle density, the perimeter of scanned images of each histological section was delimited using a photo editing program (Adobe Photoshop CS4) and the scale-calibrated area was measured in cm^2^. Afterward, follicular density (Fig. 1F, G, I and J) was calculated with the following formula: follicular density = number of follicles observed/area of the histological section (cm^2^).

### Population estimation

To determine the preantral follicle population, the oocyte nucleus was measured and used as a marker, as previously described (Gougeon & Chainy 1987). Then, the population was calculated using the formula N_t_ = (N_o_ × S_t_ × t_s_)/(S_o_ × d_o_), where N_t_ = total calculated number of follicles of a class; N_o_ = number of follicles observed in the whole ovary; S_t_ = total number of ovarian sections made; t_s_ = thickness of each ovarian section (µm); S_o_ = total number of sections evaluated; and d_o_ = mean oocyte nucleus diameter of each follicle class (Gougeon & Chainy 1987, Gastal *et al.* 2017*b*). Counting and classification of preantral follicles were performed by K A Alves, while follicle spatial distribution and population were calculated by B G Alves.

### Statistical analysis

Statistical analyses were performed using Sigma Plot, version 11.0 (Systat Software Inc., EUA). Data that were determined to be non-normally distributed using the Kolmogorov-Smirnov test (distance from geometric center, density, and population) were transformed using base 10 logarithm (Log_10_). One young mare (#2; Table 1) was found to be a statistical outlier in a few end points. This finding is relatively common in nature due to high incidences of follicular heterogeneity and individual variation; thus, this mare was intentionally kept in the data set to model what is naturally observed in several mammalian species (mare: Alves *et al.* 2016; woman: Kristensen *et al*. 2011; cow: Silva-Santos *et al.* 2011; doe: Brandão *et al.*2018; deer: Gastal *et al.* 2017*b*; mouse: Malki *et al.*2015). However, to account for the outlier status of this mare’s data, transformations or rank-based statistical tests were used. To compare mean values between groups for the follicle end points morphology, classification, distance from geometric center, density, and population, a two-way ANOVA followed by *post hoc* Tukey’s test, *t*-test, or Wilcoxon–Mann–Whitney test were used. Percentages of morphologically normal preantral follicles were assessed using chi-square or Fisher’s exact tests. Data are presented as number, mean ± s.e.m., and percentage. Statistical significance was defined as *P* < 0.05 (two-sided), and *P ≥*0.05 and ≤ 0.1 indicated a tendency to differ.

**Table 1:** Number of normal and abnormal preantral follicles observed for each classification in collected ovarian tissue per mare. A total of 438 histological sections were read (55 ± 3.1 sections per ovarian pair of each mare). Mares 1, 2, 3, and 4 were 8, 4, 4, and 9 years old, respectively. Mares 5–8 were all ≥20 years old.

Animal	Primordial		Transitional		Primary		Secondary		Overall	
	Normal	Abnormal	Normal	Abnormal	Normal	Abnormal	Normal	Abnormal	Normal	Abnormal
1	307	17	35	3	6	0	5	0	353	20
2	2679	764	3777	866	2486	419	3	1	8945	2050
3	247	34	147	13	55	2	3	0	452	49
4	233	49	60	8	20	0	2	0	315	57
5	468	6	116	7	14	3	2	0	600	16
6	81	4	32	2	10	2	2	1	125	9
7	58	3	2	4	3	1	1	0	64	8
8	56	5	16	4	7	3	0	0	79	12
Overall	4129	882	4185	907	2601	430	18	2	10,933	2221

## Results

### Follicular morphology among follicular classes

**Figure 2:**
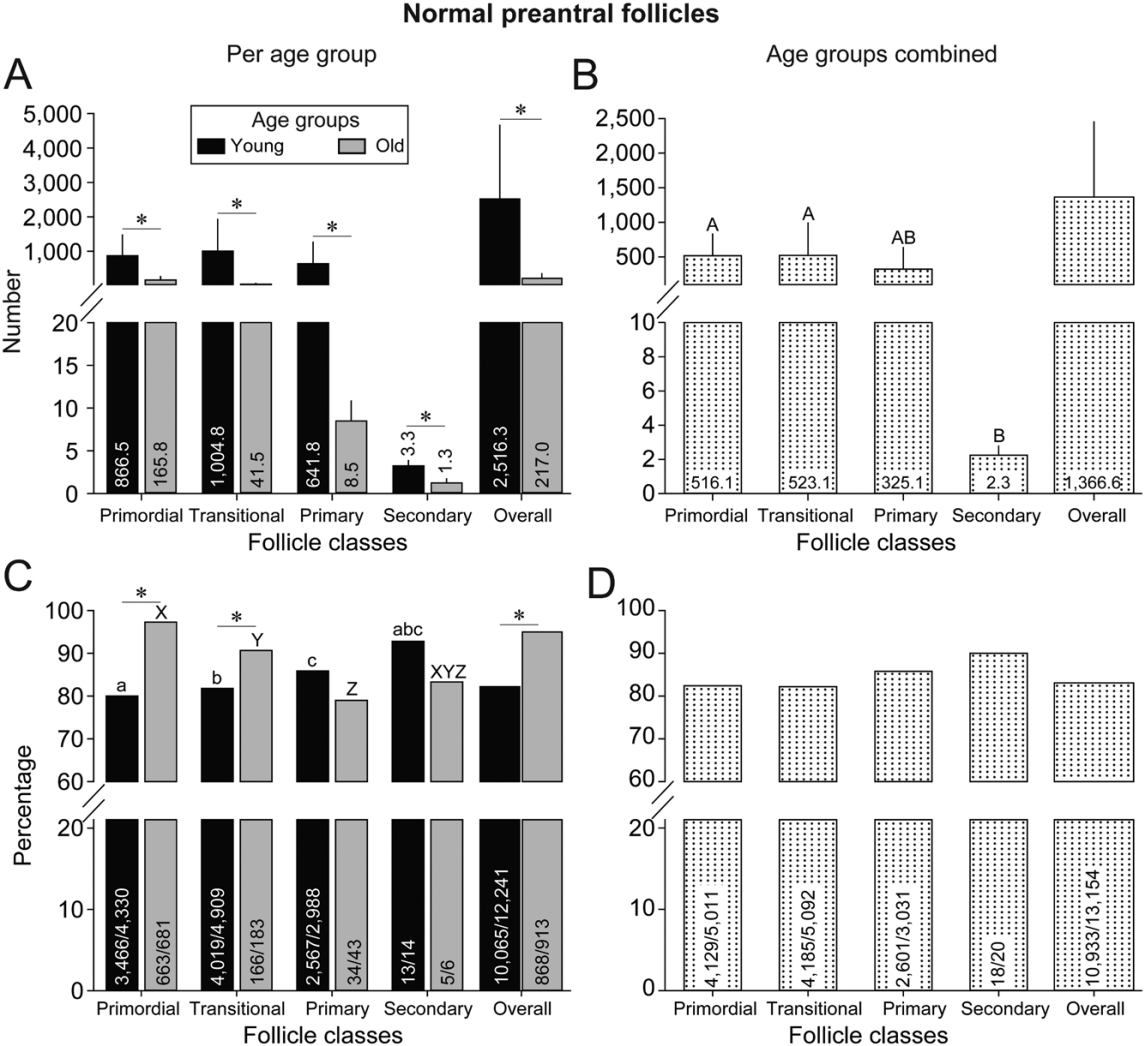
(A and B) Mean (± s.e.m.) number of preantral follicles counted and (C and D) percentage of normal preantral follicles observed in (A and C) young and old mares and (B and D) with age groups combined. *Indicates that within the same follicle class, values between age groups differed (*P*< 0.05). ^A,B^Regardless of mare age, values between follicle classes without a common superscript differed (*P* < 0.05). ^a,b,c^Within the young age group, values without a common superscript differed (*P* < 0.05). ^X,Y,Z^Within the old age group, values without a common superscript differed (*P*< 0.05). No difference (*P* > 0.05) was observed for the percentage of normal follicles between classes. (A) Below the break, Y-axis scale is every 5, and above the break, Y-axis scale changes to every 1000. (B) Below the break, Y-axis scale is every 2, and above the break, Y-axis scale changes to every 500. (C and D) Y-axis scale remains the same below and above the break.

A total of 438 ovarian histological sections were evaluated (27 ± 1.2 sections per ovary) and 13,154 preantral follicles were recorded in the lateral (*n*  = 6399) and intermediary (*n*  = 6755) ovarian portions. The number of preantral follicles (Table 1) observed per follicular class and morphological classification for each individual mare are shown. The overall number of preantral follicles observed per mare revealed a wide range (normal: 64–8945; abnormal: 8–2050); mare #7 had the lowest number of both normal and abnormal follicles, while mare #2 showed the highest. The mean (± s.e.m.) number and percentages of normal follicles (Fig. 2) per follicular class were also demonstrated between age groups and overall. Young mares had more (*P* < 0.05; Fig. 2A) normal follicles than old mares, regardless of follicular class (primordial, transitional, primary, secondary) and overall. Considering the mean number of normal preantral follicles regardless of age group (Fig. 2B), fewer (*P* < 0.05) normal secondary follicles were observed compared to early preantral follicular classes (i.e. primordial and transitional). No differences (*P* > 0.05) were observed between the mean number of primary follicles compared to the other follicular classes. Concerning the percentage of preantral follicles between age groups (Fig. 2C) in young mares, the percentage of normal follicles increased (*P* < 0.05) progressively until the primary follicular class. In contrast, old mares followed an opposite trend, whereby the percentage of normal follicles decreased (*P* < 0.05) until the primary follicular class. When comparing age groups, old mares had higher (*P* < 0.05) percentages of normal primordial, transitional, and overall follicles than young mares. Meanwhile, when age groups were pooled, the percentage of normal follicles (Fig. 2D) did not differ (*P* > 0.05).

### Preantral follicle distance from the ovarian geometric center

**Figure 3:**
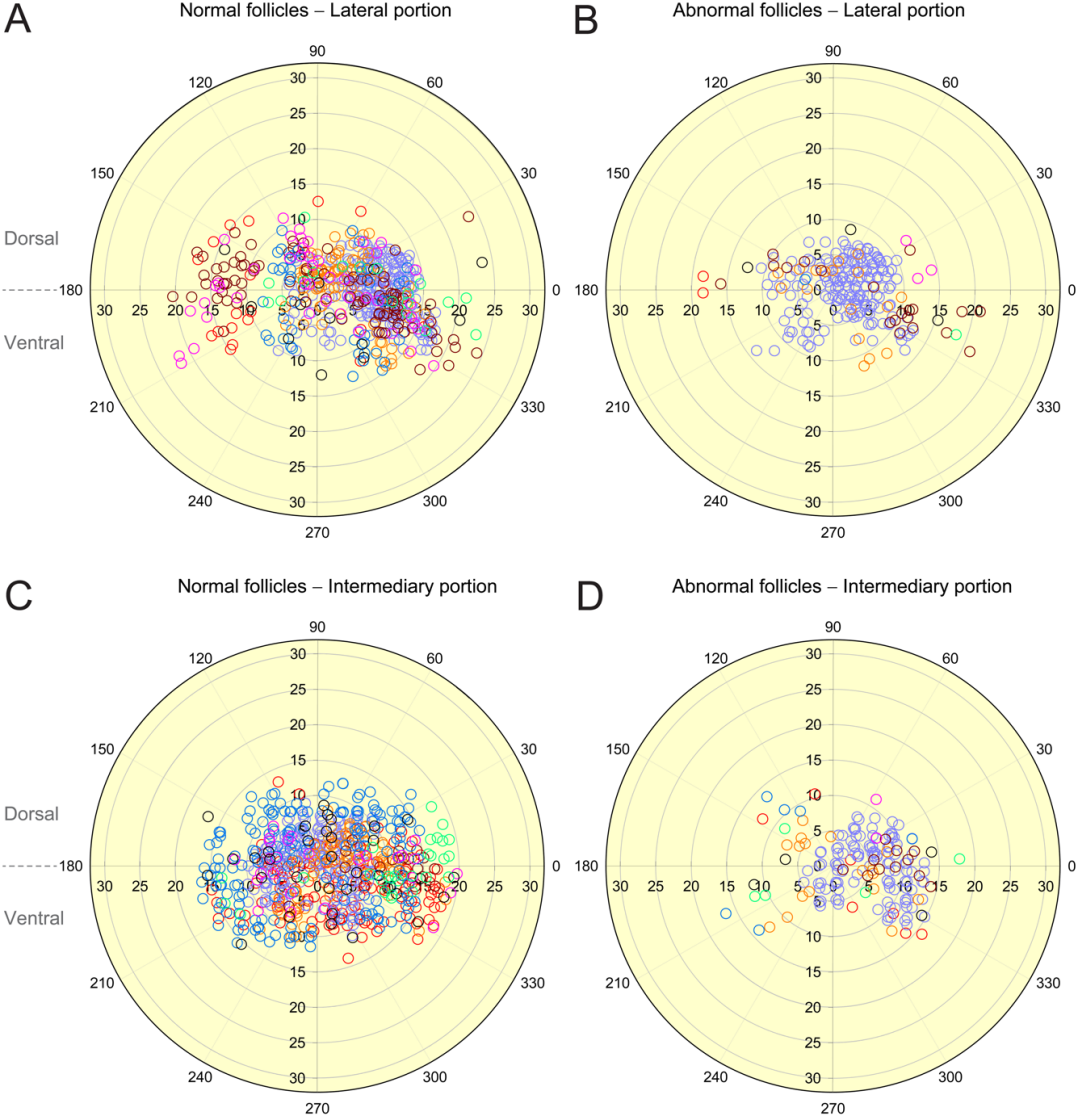
Polar plots depicting distance from the ovarian geometric center of individual follicles separated by morphological classification as (A and C) normal and (B and D) abnormal and ovarian portions as (A and B) lateral and (C and D) intermediary. Follicles from each mare (*n*  = 8) are indicated by different colors. Polar coordinates (r, θ) were determined using the distance from the geometric center (mm; r) and angulation data (°; θ) of the follicles recorded in ovarian maps. Only histological sections with clear borders and intact ovulation fossa without lacerations were chosen to be used for ovarian mapping (*n*  = 5 maps per portion; 15 maps per ovary; 240 maps total for all 16 ovaries from 8 mares). Ovarian regions were determined based upon the 180° midline; the region above is dorsal (180°–0°), and the region below is ventral (181°–360°).

A total of 240 ovarian maps were evaluated (15 per ovary), and 9284 preantral follicles were recorded in the lateral (*n*  = 4901) and intermediary (*n*  = 4383) portions and the dorsal (*n*  = 5374) and ventral (*n*  = 3910) regions. The mean distance of the observed preantral follicles in relation to the ovarian geometric center was determined considering the different ovarian portions and regions for both normal and abnormal follicles (Table 2). With regard to morphological classification (i.e. normal vs abnormal), the normal follicles within the lateral portion and ventral region were closer (*P* < 0.05) to the geometric center than were the abnormal follicles within the same portion and region. Meanwhile, in the intermediary portion, the normal follicles within both regions were farther (*P* < 0.05) from the geometric center compared to the abnormal follicles. When ovarian regions were combined, the normal and abnormal follicles in the lateral portion did not differ (*P* > 0.05) in distance from the geometric center; however, in the intermediary portion, normal follicles were farther (*P*< 0.05) than abnormal follicles from the geometric center. Within the whole ovary, normal and abnormal follicles did not differ (*P* > 0.05) regarding distance from the geometric center. Follicles in the dorsal region were closer (*P*< 0.05) to the geometric center than were follicles in the ventral region, regardless of ovarian portion and morphological classification. When comparing ovarian portions within the dorsal region, follicles in the lateral portion were farther (*P* < 0.05) from the geometric center than were those in the intermediary portion, regardless of follicular morphology. However, in the ventral region and in the regions combined, only normal follicles in the lateral portion were closer (*P* < 0.05) to the geometric center. Representative polar plots (Fig. 3) considering the distance of normal (Fig. 3A and C) and abnormal (Fig. 3B and C) follicles in regard to the geometric center in the lateral (Fig. 3A and B) and intermediary (Fig. 3C and D) ovarian portions are shown. All mares (*n*  = 8) were considered for each polar plot, with each mare represented using a different color.

**Table 2:** Mean (± s.e.m.) distance to the geometric center (mm) of normal and abnormal equine preantral follicles according to different ovarian portions (lateral and intermediary) and regions (dorsal and ventral).

Ovarian regions	Ovarian portion^§^					
	Lateral		Intermediary		Combined portions	
	Normal	Abnormal	Normal	Abnormal	Normal	Abnormal
Dorsal (*n* = 5374)^ †^	5.54 ± 0.05^aAX^	5.38 ± 0.11^aAX^	5.16 ± 0.05^aAY^	4.68 ± 0.14^bAY^	5.36 ± 0.04^aA^	5.15 ± 0.08^aA^
Ventral (*n* = 3910)	5.60 ± 0.08^aBX^	6.01 ± 0.19^bBX^	7.22 ± 0.08^aBY^	6.07 ± 0.14^bBX^	6.37 ± 0.06^aB^	6.04 ± 0.12^aB^
Combined regions	5.57 ± 0.04^aX^	5.61 ± 0.10^aX^	6.01 ± 0.05^aY^	5.37 ± 0.10^bX^	5.78 ± 0.03^a^	5.52 ± 0.07^a^

^§^Ovaries were divided longitudinally into three portions: two lateral and one intermediary. Five representative ovarian maps were evaluated per ovarian portion (*n*  = 15 maps per ovary, *n*  = 240 maps in total). Only histological sections with clear borders and intact ovulation fossa, without lacerations, were chosen for ovarian mapping. ^†^Number of preantral follicles of all classes (primordial, transitional, primary, secondary) evaluated per ovarian region. ^a,b^Between morphological classifications and within each ovarian portion and region, values without a common superscript differed (*P* < 0.05). ^A,B^Between ovarian regions and within each ovarian portion and morphological classification, values without a common superscript differed (*P* < 0.05). ^X,Y^Between ovarian portions and within each ovarian region and morphological classification, values without a common superscript differed (*P* < 0.05).

### Preantral follicle density considering different ovarian portions, regions, and age groups

**Figure 4:**
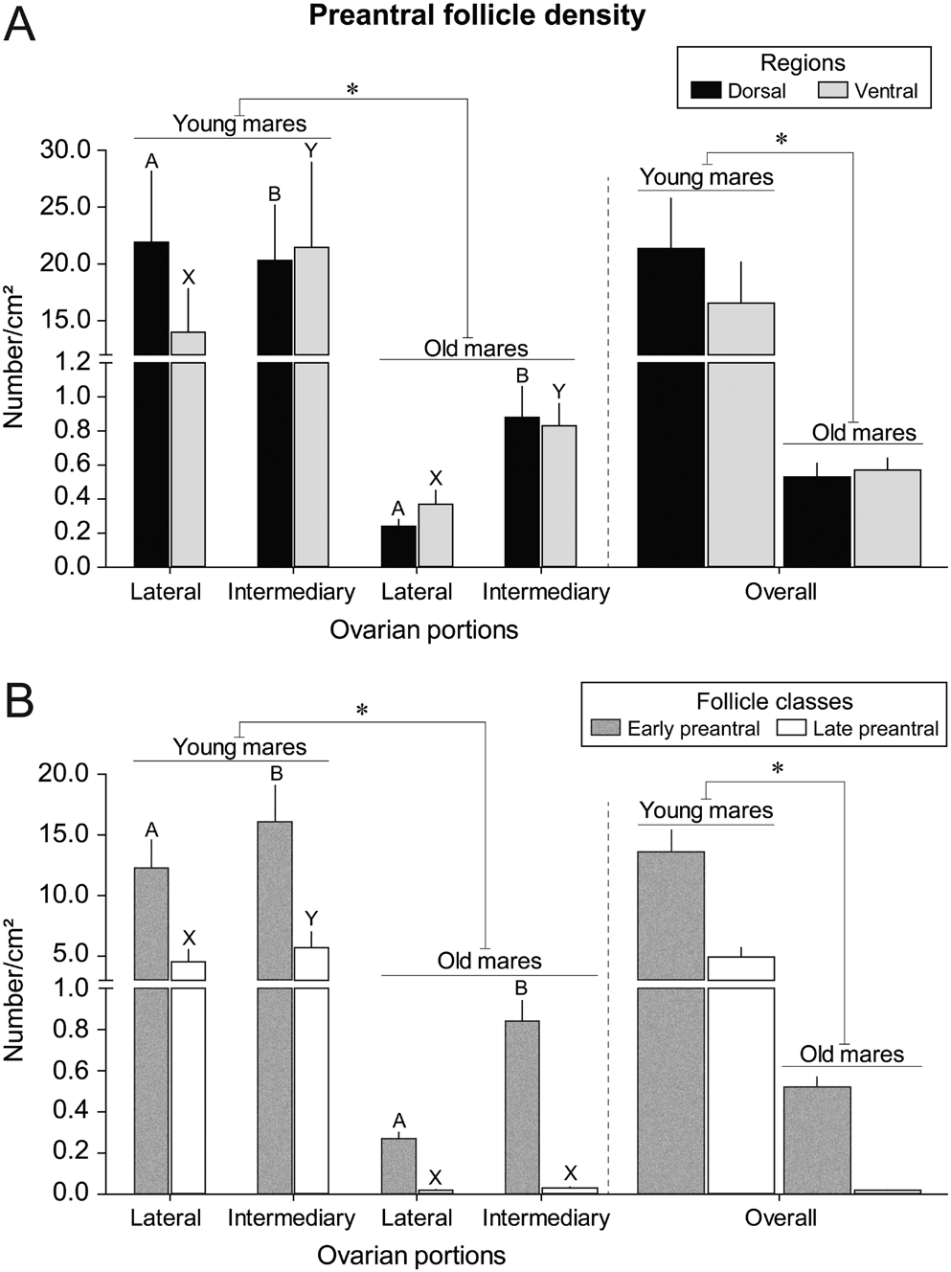
(A) Mean (± s.e.m.) preantral follicle density, regardless of follicle class, observed in different ovarian portions (lateral and intermediary), and overall, within regions (dorsal and ventral) in young (4–9 years) and old (≥20 years) mares. (B) Mean (± s.e.m.) preantral follicle density per follicle class (early preantral follicles: primordial and transitional vs late preantral follicles: primary and secondary) observed in each ovarian portion, and overall, in young (4–9 years) and old (≥20 years) mares, regardless of ovarian region. The ovaries were divided longitudinally into three portions: two lateral and one intermediary, for a total of 438 histological sections (*n*  = 277 lateral sections, *n*  = 161 intermediary sections). *Indicates young mares had greater mean follicle density than old mares within the same ovarian portion and overall. ^A,B^Within the (A) dorsal region or (B) early preantral follicle class, and within the same age group, values without a common superscript between ovarian portions differed (*P* < 0.05). ^X,Y^Within the (A) ventral region or (B) late preantral follicle class, and within the same age group, values without a common superscript between ovarian portions differed (*P* < 0.05). In the overall analyses, within each age group, the follicle densities between (A) ovarian regions and between (B) follicle classes did not differ (*P* > 0.05) when the lateral and intermediary ovarian portions were combined. No differences (*P* > 0.05) were observed in follicle densities between (A) regions and (B) follicle classes within the same portion and age group. (A and B) Below the break, Y-axis scale is every 0.2, and above the break, Y-axis scale changes to every 5.

The mean density of preantral follicles according to mare age evaluated in different ovarian portions throughout the ovarian regions and follicle class is shown (Fig. 4). Within the same ovarian portion and between age groups (Fig. 4A), young mares had higher (*P* < 0.05) densities of preantral follicles than old mares, regardless of region of the ovary. Additionally, when the lateral and intermediary portions were combined (overall analysis), young mares also showed greater (*P* < 0.05) follicular densities than old mares, regardless of ovarian region. In the dorsal regions of young mares, a higher (*P* < 0.05) density of preantral follicles was observed in the lateral portion compared to the intermediary portion; however, an opposite trend was observed in the old mares with higher (*P*< 0.05) densities in the intermediary portion. In the ventral regions of both young and old mares, the intermediary portion had higher (*P* < 0.05) follicle densities compared to the lateral portion. Furthermore, the mean preantral follicle densities according to different follicular classes and age groups were evaluated in different ovarian portions (Fig. 4B). For statistical purposes, primordial and transitional follicles were combined (early preantral), as were primary and secondary follicles (late preantral). Regardless of portions and follicular classes, as well as in the overall analysis, young mares had higher (*P* < 0.05) densities of follicles when compared to old mares. For the young mares, the intermediary portion had higher (*P* < 0.05) densities for both follicular classes than the lateral portion, while old mares showed higher (*P* < 0.05) density of early preantral follicles only in the intermediary portion. As expected, regardless of age group and ovarian portion, the densities of early preantral follicles were higher (*P*< 0.05) than those of late preantral follicles.

### Preantral follicle population according to age group and follicular classes

The mean population of preantral follicles between young and old mares, considering different follicular classes, is shown (Table 3). Primordial and transitional follicles were combined (early preantral) for statistical purposes. Within the same follicular class, the population of early preantral follicles in young mares was higher (*P* < 0.05) than in old mares; however, for late preantral follicles (primary and secondary), no differences (*P* > 0.05) were observed between ages. Furthermore, regardless of class, the overall follicular population in young mares was greater (*P* < 0.05) than in old mares. Within each age group, and when ages were combined, the follicular population decreased (*P* < 0.05) considering more advanced classes.

**Table 3:** Mean (± s.e.m.) equine preantral follicular population estimated per ovary according to age group and follicular class.

Age group (years)	Early preantral^†^	Primary	Secondary	Overall
4–9				
Mean ± s.e.m.	110,846.6 ± 63,360.1^aA^	42,019.5 ± 35,104.7^bA^	230.6 ± 83.1^cA^	152,663.5 ± 96,345.9^A^
Range	5307–489,793	112–285,264	0–580	5778–773,091
≥20				
Mean ± s.e.m.	11,038.2 ± 4688.1^aB^	628.3 ± 147.7^bA^	100.5 ± 54.8^cA^	11,749.8 ± 4802.3^B^
Range	1363–38,556	112–1,075	0–377	1477–39,874
Combined ages				
Mean ± s.e.m.	60,942.4 ± 33,284.7^a^	21,323.9 ± 17,779.3^b^	165.5 ± 50.9^c^	82,206.6 ± 50,022.4
Range	1363–489,793	112–285,264	0–580	1477–773,091

^A,B^Within the same follicular class, and overall, values without a common superscript differed (*P* < 0.05). ^a,b,c^Within the same age group, and combined, values without a common superscript differed (*P* < 0.05). ^†^Data from primordial and transitional follicles were combined. Four mares were included in each age group (eight mares total).

### Follicle population considering age groups, ovarian portions, and follicular classes

**Figure 5:**
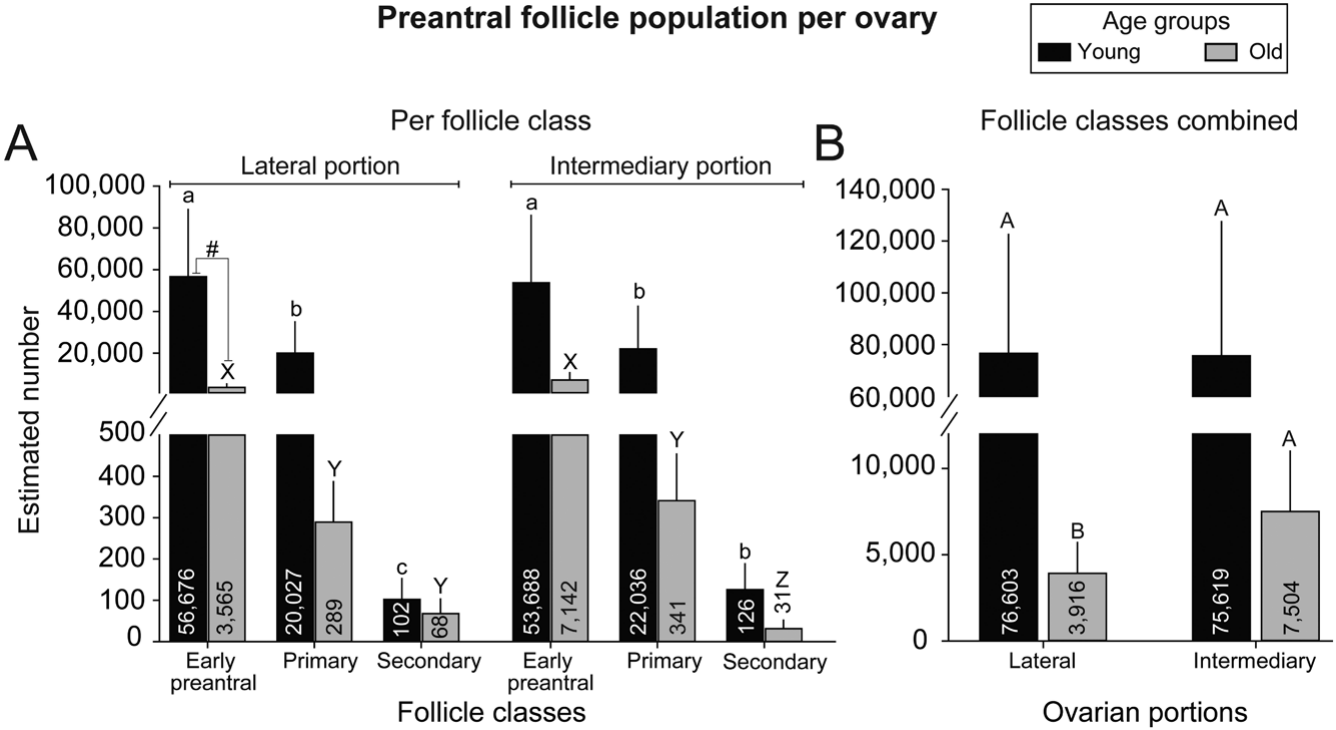
Mean (± s.e.m.) equine preantral follicle population estimated in different ovarian portions (lateral and intermediary) (A) per follicle class and with (B) follicle classes combined. ^a,b,c^Within the young age group and same ovarian portion, values without a common superscript differed (*P* < 0.05) between follicle classes. ^X,Y,Z^Within the old age group and same ovarian portion, values without a common superscript differed (*P* < 0.05) between follicle classes. ^#^Indicates tendency (*P* = 0.06) to differ between age groups within ovarian portions and follicular classes; no further differences (*P* > 0.05) were observed. No differences (*P* > 0.05) were observed between portions within each age group and the same follicular class. ^A,B^When follicle classes were combined, within each ovarian portion, values without a common superscript differed (*P* < 0.05). (A) Below the break, Y-axis scale is every 100, and above the break, Y-axis scale changes to every 20,000. (B) Below the break, Y-axis scale is every 5,000, and above the break, Y-axis scale changes to every 20,000.

The populations of preantral follicles between age groups, ovarian portions, and follicular classes are shown (Fig. 5), with primordial and transitional follicles combined (early preantral; Fig. 5A). With respect to the ovarian portions, a different pattern of follicle population was observed according to the age groups. In young mares, the lateral portion showed decreasing (*P* < 0.05) follicular populations between every follicle class, from early preantral to advanced classes. In the intermediary portion of young mares, the population decreased (*P* < 0.05) between early preantral and late preantral classes but lacked statistical difference (*P* > 0.05) between the primary and secondary classes. Interestingly, old mares showed the opposite pattern, with a steady population (*P* > 0.05) between primary and secondary classes in the lateral portion and a constant decrease (*P* < 0.05) between every follicle class in the intermediary portion. When comparing follicular populations between age groups, only the lateral portion of young mares tended (*P* = 0.06) to have a higher early preantral follicle population. Once follicle classes were combined (Fig. 5B), the population in the lateral portion was greater (*P* < 0.05) in young mares than in old mares; nevertheless, no difference (*P* > 0.05) was observed within the intermediary portions, potentially due to the variability between individuals demonstrated by the large error bars.

## Discussion

The present study quantified, for the first time in mares, the preantral follicle population including follicles smaller than 50 µm in diameter (i.e. primordial, transitional, and primary) via histological and mathematical methods. Another original aspect of this work was the evaluation of the spatial distribution of preantral follicles considering morphology and the influences of mare age. The main findings of the present study demonstrated that mares have a preantral follicle population higher than previously reported (Driancourt *et al*. 1982) and similar to that of other livestock species and women. Another novel finding demonstrated that the follicular spatial distribution within the portions and regions of the ovary changes depending upon follicle morphology and class and mare age. Finally, large individual differences (variation between mares) and heterogeneity (variation between samples from the same mare) in follicle population and spatial distribution were observed.

In the present study, the overall preantral follicle population per ovary, regardless of age groups, had a higher-than-expected population compared to a previous classical report (35,000 follicles in Driancourt *et al.* 1982 vs 82,000 in the present study). We believe this discrepancy in follicle population is due to the fact that Driancourt *et al*. (1982) counted only follicles that were greater than 50 µm in diameter. Importantly, equine primordial, transitional, and primary follicles have diameters smaller than 50 µm and compose a large percentage of the follicular reserve (Haag *et al.* 2013, Alves *et al.* 2015). For all mares in the current study, the highest numbers of follicles observed belonged to the primordial (5011 follicles, 38%) and transitional (5092 follicles, 39%) classes, and these numbers (from a total of 13,154 follicles) were included to mathematically estimate the overall ovarian population. Therefore, Driancourt *et al.* (1982) may have underestimated the follicular population of the mare by at least 50%. Another novel finding in this study was that young mares had a greater preantral follicle population (152,664 follicles) than old mares (11,750 follicles), a characteristic that is similar to other species (women: Gougeon & Chainy 1987, Gleicher & Barad 2011; bovine: Malhi *et al.* 2005; macaques: Nichols *et al.* 2005; deer: Gastal *et al.* 2017*b*). In addition to the updated equine preantral follicle population reported in the current study (82,206 ± 50,022), a large variation in the population of follicles per ovary was calculated (range: 1466–773,091). This large variation is comparable to findings in other species such as cows (ranges: 59,798–78,820, Lucci *et al*. 2002; 39,438–89,577, Silva-Santos *et al.* 2011), ewes (range: 7333–44,633, Amorim *et al*. 2000), gilts (range: 67,599–291,898, Alves *et al*. 2012), does (range: 20,122–80,739, Lucci *et al.*1999), and women (range: 2700–79,600, Gougeon & Chainy 1987). This fact leads us to assume that the mare, despite having some unique anatomical differences (i.e. a single point where all ovulations occur called the ovulation fossa and inverted follicular layers where the ovarian cortex makes up the center of the ovary while the medulla surrounds the cortex) compared to other species, could continue to be an alternative animal model for dual-purpose, dual-benefit studies considering ARTs (Benammar *et al*. 2021).

An interesting effect of age on the populations of preantral follicles in the intermediary and lateral portions was found. To the best of our knowledge, there is no report in the literature that has evaluated the population of preantral follicles regarding spatial distribution in any species. The reason for the different patterns in the populations of preantral follicle classes between portions within each age group observed in this study is unknown and deserves further investigation.

In the present study, large individual differences and follicular heterogeneity were characterized for each end point evaluated (i.e. follicle number, distance from geometric center, density, and population). Extensive follicular heterogeneity has been reported in mares (Alves *et al*. 2017, 2018, Gonzales *et al.*2017) as well as other livestock species (cows: Aerts *et al*. 2008, Silva-Santos *et al.*2011; ewes: Fransolet *et al*. 2014) and women (Fabbri *et al*. 2012). This follicular heterogeneity and large differences between individuals and tissue samples may be due, at least in part, to the working hypothesis of ovarian plasticity (Woodruff & Shea 2011, Alves *et al.* 2018), which hypothesizes that preantral follicles migrate within the ovarian cortex during early folliculogenesis. Indeed, folliculogenesis encompasses a dynamic and complex process characterized by follicular quiescence (Faire *et al.*2015), activation, growth (Gaytan *et al.*2015), tissue remodeling of the ovarian stroma by antral follicles (Riley *et al.*2001), migration of preovulatory follicles toward the ovulation fossa (for review, see Gastal 2011) or atresia (Tatone *et al.* 2008). Altogether, the aforementioned events may lead to a heterogeneous and widely variable follicle population, both between and within individuals.

A core novel finding of the present study showed that follicle distance from the geometric center of the ovary in different portions and regions changes depending on morphology. While all follicles, regardless of morphology, within the dorsal region are closer to the geometric center than in the ventral region, morphologically normal follicles within the dorsal region and intermediary portion are farther from the geometric center than are abnormal follicles. These findings fit well into the working hypothesis proposed by Alves *et al*. (2018) that, as preantral follicles develop until the primary classification, they migrate closer to the geometric center. Once these follicles develop into secondary follicles, they begin to migrate farther from the geometric center. Thus, we hypothesize that, in the intermediary portion, once preantral follicles become abnormal, the migration process stops closer to the geometric center, while normal follicles will continue to migrate farther from the geometric center. These normal preantral follicles will later form the antral follicles randomly distributed throughout the ovarian cortex (Kimura *et al*. 2009, Alves *et al.*2018). This migration is potentially necessary for the continuation of folliculogenesis during the critical transition from preantral to antral follicle classes. To this end, studies that simultaneously evaluate the distance from the geometric center of follicles of differing classes in combination with morphology are warranted to validate our current working hypothesis.

The number of normal preantral follicles in the present study, as expected, was higher in young mares, regardless of class. This result is in accordance with previous reports, as aging is associated with significant decreases in the number of preantral follicles observed in the ovaries of livestock species (equine: Haag *et al.* 2013, Alves *et al.* 2017; bovine: Malhi *et al.* 2005), non-human primates (Nichols *et al.* 2005), and women (Gleicher & Barad 2011). Interestingly, despite having lower numbers of normal primordial and transitional follicles, old mares had higher percentages of these follicles than young mares in this study. These findings may be explained due to the fact that, during the lifespan of a female, a majority of follicles will begin to develop and leave the pool of quiescent follicles (Tatone *et al.* 2008). This process is potentially more intense in young females because of the large number of follicles that are available to grow. Once follicular growth has begun, only a tiny percentage of these preantral follicles will reach ovulation, with the rest undergoing atresia, decreasing the number of follicles within the ovary (Tatone *et al.* 2008) as a mare ages. The follicles that do remain in quiescence (primordial) or have likely started to grow (transitional) are potentially more resistant to atresia, as suggested by Aguiar *et al.* (2017). Thus, we speculate that, once a mare reaches old age, only the remaining primordial and transitional follicles are more often morphologically normal.

In terms of follicular density, the present study found that there is an age effect on the densities of preantral follicles in the different portions of the equine ovary. As expected, young mares had higher follicle densities for all ovarian portions and regions than old mares; however, different trends between portions were observed within age groups and regions. In this regard, the intermediary portion of old mares displayed similar follicle density between regions; meanwhile, a higher density of the early preantral follicle class was observed. Therefore, we hypothesize that the few remaining, atresia-resistant early preantral follicles of old mares tend to reside in the intermediary portion of the ovary, regardless of ovarian region. This lack of regional difference could potentially be explained by the fact that preantral follicles exhibit a more dispersive pattern as a mare reaches older age (Alves *et al.* 2018). Our hypothesis is further supported by the concept of ovarian plasticity (Woodruff & Shea 2011), as these early preantral follicles are still migrating toward the center of the ovary located in the intermediary portion. Regarding young mares, we postulate that the different trends of follicle density within the ovarian regions and higher densities of both follicle classes in the intermediary portion are potentially due to higher levels of follicular activity. For ovulation to occur in the mare, large antral/preovulatory follicles must develop and migrate toward the ovulation fossa within the ventral region of the ovary (Riley *et al*. 2001, Gastal 2011). Due to these facts, we hypothesize that, as these large follicles migrate toward the ovulation fossa, the much smaller preantral follicles are pushed into the dorsal region of the lateral portion in young mares. However, the intense follicular activity of young mares, translated by high numbers of early preantral and primary follicles migrating toward the ovarian geometric center (Alves *et al.* 2018), is potentially reflected in this study by the higher preantral follicle density in the ventral region of the intermediary portion. Considering that young mares have higher numbers of both preantral and antral follicles than old mares (Ginther *et al.*2008, 2009, Alves *et al.* 2017, Goncalves *et al*. 2020), our hypotheses may explain the different trends in follicular density observed in this study. In this aspect, future studies assessing follicle density of different classes, particularly late secondary and early antral follicles, in combination with morphology, portion, and regional location within the ovary, are appealing.

Overall, the present study reports for the first time (i) a higher preantral follicle population than originally reported by Driancourt *et al.* (1982), (ii) an effect of mare age on the spatial distribution of morphologically normal and abnormal follicles, (iii) an age effect on the density of follicular classes, and (iv) an effect of age on the population distribution of follicle classes. The in-depth characterization of the distribution and population of preantral follicles in the mare ovary provided by this study can aid in improving reproductive studies, ARTs, and procedures regarding mechanisms involving ovarian plasticity and follicular migration. Therefore, the application of results from this study may assist in targeting certain areas of the equine ovary to obtain higher follicular densities with better quality (i.e. morphologically normal) and particular classes. For example, if preservation of primordial follicles is desired in young equine ovarian tissue, the intermediary portion and ventral region of the ovary should be targeted for ovarian biopsy to harvest a high density of normal primordial follicles. Furthermore, using the information reported by the current study, we have provided working hypotheses that should be further explored to elucidate mechanisms related to ovarian physiology, folliculogenesis, and follicular migration.

## Declaration of interest

The authors declare that there is no conflict of interest that could be perceived as prejudicing the impartiality of the research reported.

## Funding

This work was supported by Southern Illinois University (SIU), Carbondale, IL, USA. K A Alves and G D A Gastal were recipients of PhD scholarships and B G Alves was the recipient of a post-doctoral fellowship from the National Council for Scientific and Technological Development (CNPq). Funders had no role in study design, data collection and analysis, decision to publish, or preparation of this manuscript.

## Author contribution statement

Conceived and designed the experiment: B G A, K A A, G D A G, and E L G. Performed the experiment: B G A, K A A, G D A G, and E L G. Analyzed the data and prepared figures: K A H, F L N A, B G A, K A A, M O G, and E L G. Contributed reagents/materials/analysis tools: E L G. Wrote the paper: K A H, F L N A, and E L G.
